# Effects of repetitive transcranial magnetic stimulation on prefrontal cortical activation in children with attention deficit hyperactivity disorder: a functional near-infrared spectroscopy study

**DOI:** 10.3389/fneur.2024.1503975

**Published:** 2024-12-06

**Authors:** Jing Wang, Zhuo Zou, Haoyu Huang, Yangping Zhang, Xuemei He, Hang Su, Wenjuan Wang, Yingjuan Chen, Yun Liu

**Affiliations:** Department of Rehabilitation, Kunming Children's Hospital, Kunming Medical University, Kunming, Yunnan, China

**Keywords:** attention-deficit/hyperactivity disorder, repetitive transcranial magnetic stimulation, near-infrared functional brain imaging, prefrontal cortex, cortical activation

## Abstract

**Background:**

Attention deficit hyperactivity disorder (ADHD) is a prevalent neurodevelopmental disorder characterized by inattention, impulsivity, and hyperactivity. With the continuous development of neuromodulation technology, Repetitive Transcranial Magnetic Stimulation (rTMS) has emerged as a potential non-invasive treatment for ADHD. However, there is a lack of research on the mechanism of rTMS for ADHD. Functional near infrared spectroscopy (fNIRS) is an optical imaging technique that reflects the brain function by measuring changes in blood oxygen concentration in brain tissue. Consequently, this research utilized fNIRS to examine the impact of rTMS on the core symptoms and prefrontal cortex activation in children with ADHD, which provides a reference for the clinical application of rTMS in the treatment of ADHD.

**Methods:**

Forty children with ADHD were chosen as research subjects and randomly assigned to two groups: a treatment group (20 subjects) and a control group (20 subjects). The control group received non-pharmacological interventions, whereas the treatment group was administered rTMS in conjunction with non-pharmacological interventions. Clinical symptom improvement was evaluated using SNAP-IV scale scores both before and after treatment. Additionally, fNIRS was utilized to monitor alterations in the relative concentrations of oxyhemoglobin (HbO_2_) and deoxyhemoglobin (HbR) in the prefrontal cortex during resting state and during the Go/no-go task state, both pre- and post-treatment.

**Results:**

In conclusion, the study comprised 17 participants in the treatment group and 18 in the control group. Initially, the SNAP-scale scores were comparable between the groups, with no significant differences observed (*p* > 0.05). Post-treatment, a notable reduction in SNAP-scale scores was evident (*p* < 0.05), with the treatment group exhibiting a more pronounced decrease (*p* < 0.05). Following the intervention, both groups demonstrated enhanced Resting-state functional connectivity (RSFC) in the prefrontal cortex, as indicated by a significant increase compared to pre-treatment levels (*p* < 0.05). Specifically, the treatment group showed superior RSFC in the left dorsolateral prefrontal cortex, right dorsolateral prefrontal cortex, left medial prefrontal cortex, and right medial prefrontal cortex compared to the control group (*p* < 0.05). However, no significant differences were noted in RSFC of the left and right temporal lobes between the two groups (*p* > 0.05). In the Go/no-go task, the treatment group recorded higher mean HbO_2_ concentrations in the aforementioned prefrontal cortical regions compared to the control group (*p* < 0.05). Conversely, no statistically significant disparities were observed in the left and right temporal lobes of both groups.

**Conclusion:**

rTMS shows promise as a treatment for ADHD by modulating prefrontal cortical activation. fNIRS provides a valuable method for assessing these effects, offering insights into the neurobiological mechanisms underlying rTMS therapy.

## 1 Introduction

ADHD is a common chronic neurodevelopmental disorder in children and adolescents, characterized by attention deficits that are inconsistent with age, often accompanied by excessive activity, impulsivity, learning difficulties, and other symptoms, affecting 8 to 12% of children worldwide (1). ADHD not only affects the development of cognitive and learning functions in affected children but may also lead to behavioral problems, affecting the children’s daily activities and social interactions, and can even result in juvenile delinquency issues (2). If ADHD is not treated promptly, more than half of the patients’ clinical symptoms will persist into adolescence, and 30% of the patients will continue to exhibit clinical symptoms into adulthood (3). ADHD is thought to arise from a complex interplay of genetic, environmental, and neurobiological factors. The etiology of ADHD is multifaceted, with genetic factors being highly significant, as indicated by family studies and twin studies suggesting a heritability rate of around 80%. Neurotransmitter imbalances, particularly involving dopamine and norepinephrine, are also implicated in the disorder’s pathogenesis. Environmental factors, such as prenatal exposure to toxins, maternal smoking, et al., may also contribute to the development of ADHD (4). Furthermore, structural and functional brain imaging studies have revealed differences in the prefrontal cortex and other areas associated with cognitive control in individuals with ADHD (5). The latest neuroimaging studies suggest that the functional or structural network organization of the brains of children with ADHD is disrupted, and dysfunction in the organization of brain network tissue and in functional connectivity may be significant causes of ADHD (6). Recent studies have shown that children with ADHD exhibit significant changes in the topological organization of brain networks compared to typically developing individuals. These changes are characterized by a decrease in the overall efficiency of the brain network and an increase in local efficiency, which may be associated with the various clinical ratings or deficits in related cognitive functions typical of ADHD (7).

As neuromodulation technology continues to evolve, rTMS has increasingly been applied to improve the clinical symptoms of children with ADHD. rTMS is a non-invasive brain stimulation technique that has been increasingly investigated for its potential therapeutic effects on cognitive functions in individuals with ADHD. rTMS is a non-invasive neuromodulation method that delivers magnetic pulses to the head through a coil, generating an electric field in the cerebral cortex. This process promotes the development of synaptic connections and the transmission of neurotransmitters, thereby modulating the excitability of neural activity. The treatment involves delivering magnetic pulses to specific regions of the brain, with the aim of modulating neural activity and improving cognitive control. Studies have shown that low-frequency rTMS treatment applied to the left dorsolateral prefrontal cortex of ADHD patients, at a stimulation intensity of 90% of the resting motor threshold (RMT), can improve symptoms such as attention, hyperactivity, and impulsivity in children (8, 9). Recent research has shown that rTMS may have a positive impact on ADHD symptoms, particularly in enhancing sustained attention and processing speed. A Meta-analysis of randomized controlled trials (RCTs), which included a total of 189 participants, demonstrated that rTMS was more effective in improving sustained attention in patients with ADHD compared to control groups (SMD = 0.54, *p* = 0.001). Additionally, rTMS showed efficacy in improving processing speed (SMD = 0.59, *p* = 0.002), but not for enhancing memory or executive function. The therapeutic effects of rTMS is believed to be related to its ability to induce brain dopamine release and increase synaptic plasticity, which are crucial for the cognitive functions typically impaired in ADHD. Studies have also suggested that rTMS may be more effective when targeting the right prefrontal cortex (PFC) for improving inattention, which aligns with the observation of right hemisphere under-activation in individuals with ADHD (10). However, there is currently a lack of research on the mechanism of action of rTMS in the treatment of ADHD. fNIRS is an optical imaging technique that reflects brain function by measuring changes in blood oxygen concentration in brain tissue. Before conducting an fNIRS examination on a subject, sensors are placed on the subject’s head. Then, near-infrared light is shone onto the brain tissue, which absorbs and scatters the light. Detectors collect the blood oxygen levels within the brain tissue, thereby calculating the functional connectivity of brain regions (11). fNIRS has often been used to explore the neural bases associated with ADHD, such as response inhibition, working memory, cognitive flexibility, attention, and emotional regulation (12). Resting state is a natural imaging paradigm, and resting-state functional near-infrared spectroscopy (rs-fNIRS) imaging is convenient to operate and easy to perform in clinical practice, especially for pediatric patients. Studies have demonstrated the feasibility and reliability of rs-fNIRS in detecting brain functional connectivity and network topological properties (13). There is also a growing number of studies applying fNIRS to explore executive dysfunction in children with ADHD, using tasks that involve response inhibition (such as the Go/No-go test) to assess executive dysfunction in these children (14, 15).

In summary, ADHD is a complex neurodevelopmental disorder with a multifactorial etiology that involves genetic predisposition, neurotransmitter imbalances, and alterations in brain structure and function. rTMS represents a promising therapeutic approach that targets the underlying neurobiological mechanisms of ADHD, and fNIRS serves as a valuable tool for assessing and monitoring the effects of rTMS on cortical activation in children with ADHD. This study aims to investigate the effects of rTMS on prefrontal cortical activation in children with ADHD using fNIRS, providing a comprehensive understanding of the therapeutic potential of rTMS and its impact on the brain function.

## 2 Materials and methods

### 2.1 Participants

The Ethics Committee of Kunming Children’s Hospital gave its approval to all study protocols and research techniques (2022-03-307-K01), ensuring that they adhered to the World Medical Association’s Declaration of Helsinki regarding the use of humans in testing. All participating children’s parents gave their informed consent, and each participant gave their written consent before the experiment began.

A total of 40 children with ADHD were recruited from Kunming Children’s Hospital and randomly assigned to the treatment group (*n* = 20) and the control group (*n* = 20). All children with ADHD were diagnosed based on the Diagnostic and Statistical Manual of Mental Disorders, Fifth Edition (DSM-5, American Psychiatric Association, 2013) by a qualified child psychiatrists with experience in ADHD (16). During the research process, 5 children were lost to follow-up, resulting in a final selection of 35 cases. Among them, the treatment group consisted of 17 cases, with 12 males and 5 females, and an average age of (8.45 ± 1.53) years; the control group had 18 cases, with 11 males and 7 females, and an average age of (8.23 ± 1.42) years. Demographic information is shown in Table 1.

**Table 1:** Demographic information of participants.

	Treatment group		Control group		χ^2^	*p*
	Mean	SD	Mean	SD		
Gender (boy/girl)	12:5		11:7		0.349	0.55
Age (years)	8.45	1.53	8.23	1.42	0.440	0.66

The inclusion criteria are as follows: ① Diagnosed with ADHD according to the DSM-5; ② Age between 6 and 12 years old; ③ Children who can cooperate with doctors for rTMS treatment and fNIRS examination; ④ Have not received medication and non-pharmacological treatments; ⑤ An intelligence quotient (IQ) of ≥80 on the Chinese version of the Wechsler Intelligence Scale for Children-Revised (WISC-CR); ⑥ No chronic physical illnesses and psychiatric disorders; ⑦ Children who can cooperate with doctors for rTMS treatment and fNIRS examination. All parents of children with ADHD completed the Chinese version of the Swanson, Nolan, and Pelham Questionnaire (SNAP-IV, 9 items for attention deficit, 9 items for hyperactivity) (17).

### 2.2 Intervention

#### 2.2.1 Control Group

Non-pharmacological interventions, include (18, 19): ① Behavioral Therapy: Step-by-step application of behavior modification and shaping techniques to intervene in problematic behaviors, including positive reinforcement, extinction, modeling, etc.; ② Applied Behavior Analysis: A structured therapeutic approach that combines cognitive strategies and behavioral techniques, aiming at correct cognitive deficits while employing behavior management techniques to improve emotional and behavioral issues, and establish new cognitive-behavioral patterns. ③ Attention Training: Mainly conducted from two aspects, visual and auditory. Visual training includes Schulte table test, dot-to-dot tracking, with the completion time of the training used as standard. Auditory training includes auditory comprehension and memory, with the correct response rate as the standard. By focusing on both visual and auditory aspects, the aim is to improve the attention levels of children with ADHD. The training principles follow an individualized, graded approach, progressing step by step. The aforementioned treatments are administered once every other day, three times a week, with a four-week period constituting one course of treatment, and the therapy continues for three consecutive courses. All non-pharmacological treatments are performed by a supervising therapist who is certified as a rehabilitation therapist and have been working for at least 5 years.

#### 2.2.2 Treatment group

**Figure 1:**
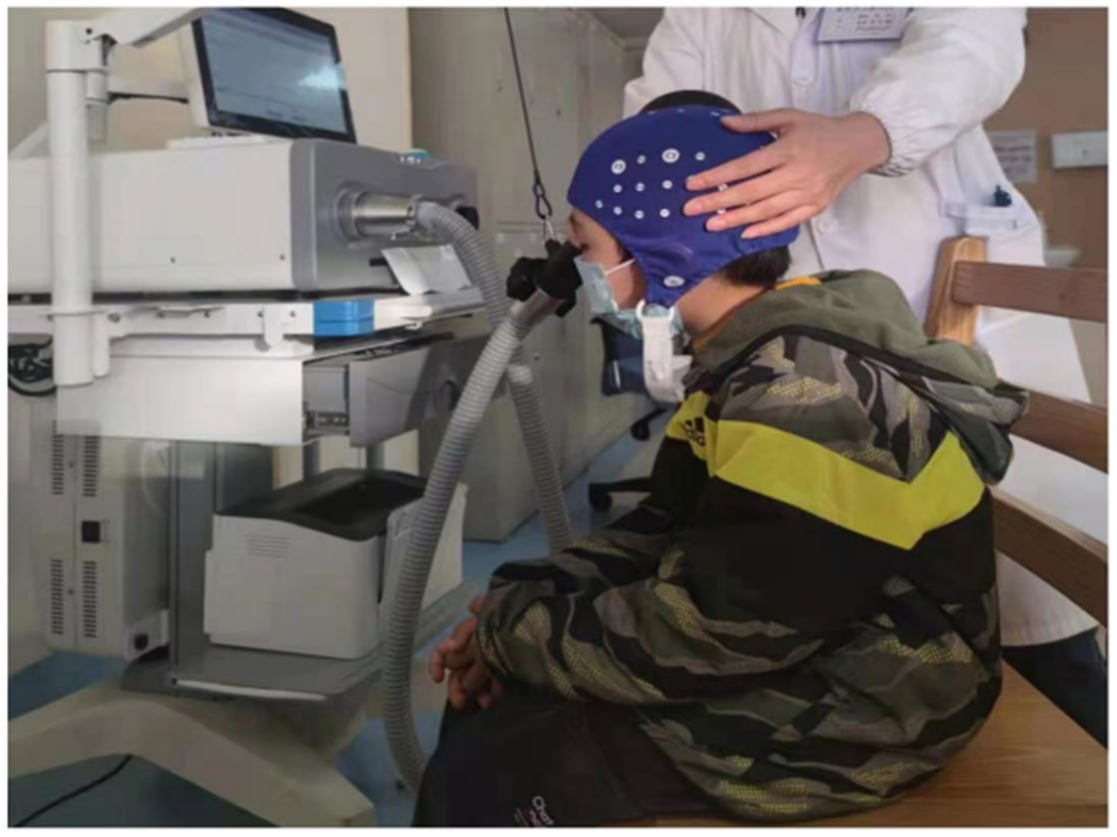
rTMS treatment.

On the basis of non-pharmacological interventions, rTMS treatment is conducted: The Magneuro 60 transcranial magnetic stimulator, produced by Nanjing Weisi Medical Technology Co., Ltd., with an “8”-shaped stimulation coil, is used. Before treatment, the child sits in a proper position and wears the positioning cap to select the single-pulse mode to stimulate the left thumb motor cortex area (M1 area). By continuously fine-tuning the magnetic stimulation site and intensity, the minimum magnetic stimulation intensity that can induce a motor evoked potential (MEP) amplitude >50 μV in at least 5 out of 10 consecutive stimuli is determined as the child’s resting motor threshold (RMT) (20). During the formal treatment, the child sits and wears a positioning cap, with the dorsolateral prefrontal cortex (DLPFC) selected as the stimulation target. The stimulation frequency is 1 Hz, stimulation intensity is 80% RMT, each sequence of stimulation lasts for 10 s, with a 5 s interval after each sequence. The total magnetic pulse quantity is approximately 1,200 times, with each treatment session lasting 20 min. The aforementioned treatment is administered once every other day, three times a week, with a four-week period constituting one course of treatment, and the therapy continues for three consecutive courses (Figure 1). rTMS is performed by physicians who have received specialized training and have been working for at least 5 years.

### 2.3 Acquisition of fNIRS data

#### 2.3.1 Resting-state data acquisition

**Figure 2:**
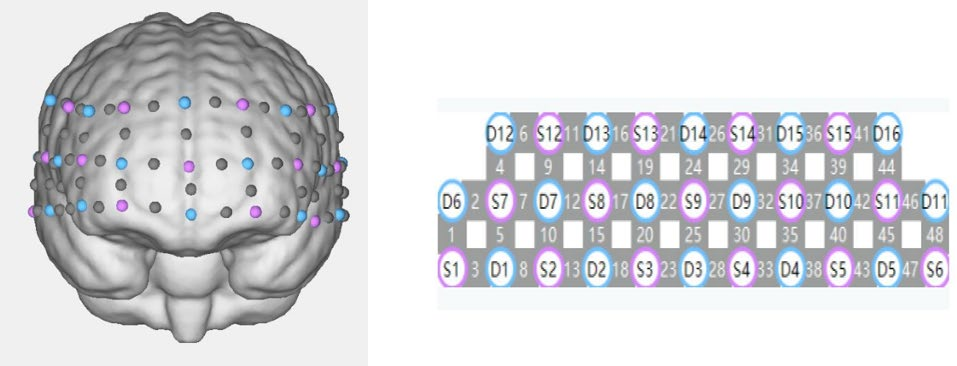
48-channel arrangement (transmitting probes in blue, receiving probes in purple).

Using the fNIRS device (NirScan, Danyang Huichuang Medical Equipment Co., Ltd., China) to detect spontaneous changes in Hb concentration in 48 channels under resting-state conditions, non-invasively measuring the concentrations of HbO_2_, HbR, and HbT through changes in light intensity. The device can be configured to a maximum of 48 effective channels, positioned according to the international 10–20 system, covering the frontal and temporal lobes (Figure 2). Based on the device’s coordinates, the 48 channels are divided into each brain region, thus allowing the selection of specific regions of interest (ROIs) in the study. The ROIs in this study include the left dorsolateral prefrontal cortex (channels CH32, 44–48), the right dorsolateral prefrontal cortex (channels CH24, 37, 38, 39, 41, 42), the left medial prefrontal cortex (channels CH9–12, 25, 27, 29–31), the right medial prefrontal cortex (channels CH4–8, 21–23, 26, 28, 40), the left temporal lobe (channels CH13, 15, 16, 33–36), and the right temporal lobe (channels CH1–3, 17–20). Before wearing, first determines the midpoint of the top of the head (Cz), which is the intersection of the line from the root of the nose to the occipital protuberance and the line connecting the bilateral external auditory meatus. After putting on the cap, adjust the probes to ensure that the position of the optode cap worn by all subjects is consistent, and start data collection after the signal is stable, scanning the brain continuously for 10 min in a resting state.

#### 2.3.2 Go/no-go task state acquisition

Go/no-go task was generated by E-Prime2.0 and presented in a 17′′ tablet computer screen. The distance between the subject’s eyes and the screen was ∼50 cm. Including the Go task and the Go/no-go task. Each group consists of alternating go (baseline) and Go/no-go (target) blocks. They were repeated six times in the order of Go and Go/no-go. Each block contained instructions for 3 s at the beginning of the task, and each condition lasted for 24 s. In the Go task, two types of stimulus images (cats and dogs) will be presented on the tablet. During the Go task phase, both stimulus images are go stimuli, and a button press response is required for both, that is, “press for both cats and dogs”. In the no-go task, two types of stimulus images (chickens and ducks) will be presented on the tablet. During the Go/no-go task phase, one type of stimulus image serves as the go stimulus (chicken), and the other as the no-go stimulus (duck). A button press response is required as quickly as possible when the go stimulus (chicken) appears, whereas no button press is needed when the no-go stimulus (duck) appears, that is, “press for chicken, not for duck”. A total of 24 stimulus images were presented for each GO or no-go task, 12 for each stimulus image, in random order, and each stimulus image was presented at 1 s intervals. At the upper end of the plate, the first 0.9 s within 1 s is the stimulus picture, and the last 0.1 s is the key feedback. All subjects received one practice session before the treatment. The reaction time (reaction time, RT) and accuracy rate were recorded for each trial (accuracy rate = The number of right responses/The total number of responses).

### 2.4 Processing and analysis of the fNIRS data

Using the NirSpark (Danyang Huichuang Medical Equipment Co., Ltd., China) analysis software for fNIRS data processing, include: ① Quality Control: Detect motion artifacts, apply filtering, and exclude substandard data through the quality control module. The unqualified data typically characterized by severe motion artifacts, low signal-to-noise ratios, or non-compliance with requirements even after band filtering. The unsatisfactory time intervals containing sudden, obvious, and discontinuous noise were excluded. ② Preprocessing: The light intensity signals from each channel are converted into HbO_2_, HbR, and HbT concentration signals. Spline interpolation is a commonly used correction method that specifically addresses pre-identified artifacts. Therefore, we applied spline interpolation to correct motion artifacts in the fNIRS data. Motion artifacts manifest as high-amplitude or high-frequency spikes caused by shifts in the optodes and scalp. Signal changes exceeding 2 standard deviations across the entire time series are considered motion artifacts. We filtered physiological noise caused by heartbeat, respiration, and other factors using a bandpass filter in the range of 0.01 Hz to 0.2 Hz. ③ According to the modified Beer–Lambert law, the original optical density values are converted into concentration changes of HbO_2_ and HbR. ④ Brain functional connectivity strength calculation: Pearson correlation is used to compute the correlation between the fNIRS signals of each channel, obtaining the Pearson correlation coefficients between the channels to construct a functional connectivity matrix. The Pearson correlation coefficients of the oxyhemoglobin concentration time series between each channel pair are calculated and subjected to Fisher-Z transformation. The transformed values, Z-scores, are defined as the functional connectivity strength values among the channels. During the Go/no-go task, the calculation of HbO_2_ concentration involves computing the average difference in HbO_2_ concentration changes for each channel during the target period (4–27 s after the start of the block), selecting regions of interest (ROIs) to average the classified channels, and conducting functional connectivity network analysis using the dorsolateral prefrontal cortex as a seed point. The difference between HbO_2_ changes and baseline during the target period is extracted, and the average HbO_2_ concentration for each group of ROIs is calculated, which serves as the primary observation indicator.

### 2.5 Statistical analysis

To better compare numerical variables between the observation and control group, the chi-square (χ2) test was used to compare the clinical characteristics and the independent samples *t*-test to compare the behavioral performance, resting-state functional connectivity, and average HbO_2_ of the two groups of children. All statistical analyses were conducted using the SPSS statistical software package (version 26.0) with a statistical threshold *p*-value of <0.05.

## 3 Results

### 3.1 Clinical characteristics

The comparison of SNAP-IV Scale (Parent Version) Scores Before and After Treatment of each study participant are presented in Table 2. Before treatment, there was no difference in SNAP-IV scale (parent version) scores between the two groups of children in terms of attention deficit scoring, hyperactivity-impulsivity scoring, and oppositional defiance scoring (*p* > 0.05). After treatment, the scores of both groups significantly decreased compared to their own pre-treatment scores (*p* < 0.05), with the treatment group scoring significantly lower than the control group (*p* < 0.05).

**Table 2:** SNAP-IV of participants.

		Treatment group		Control group		*t*	*p*
		Mean	SD	Mean	SD		
Before treatment	SNAP-IV IA	2.39	1.23	2.36	1.18	0.073	0.94
	SNAP-IV IH	1.23	0.58	1.24	0.69	0.10	0.92
	SNAP-IV ODD	1.89	1.21	1.86	1.18	0.07	0.94
After treatment	SNAP-IV IA	0.88	0.34	1.13	0.21	2.21	0.03^*^
	SNAP-IV IH	0.73	0.39	0.96	0.27	2.03	0.04^*^
	SNAP-IV ODD	0.87	0.24	1.12	0.35	2.39	0.02^*^

SD, standard deviation; SNAP-IV IA, inattention subscale scores; SNAP-IV IH, hyperactivity subscale scores; SNAP-IV ODD, oppositional defiance. ^*^ *p* < 0.05.

### 3.2 Behavioral performance

The average accuracy rates and RTs in each Go/no-go task for treatment group and control group are summarized in Table 3. The treatment group had a significantly higher accuracy rate in the no-go block than the control group (*p* < 0.05). There was no significant difference in RTs and accuracy rates in the Go block, and RTs in the no-go block between the treatment group and the control group (*p* > 0.05).

**Table 3:** Go/no-go task performance.

		Treatment group		Control group		*t*	*p*
		Mean	SD	Mean	SD		
Before treatment	Accuracy-go trail (%)	91.58	4.23	91.74	3.76	0.11	0.90
	Accuracy-no go trail (%)	72.76	5.25	74.21	5.43	0.81	0.41
	RT-go trail (ms)	378.68	32.25	379.42	32.36	0.06	0.94
	RT-no go trail (ms)	443.23	45.63	442.87	47.36	0.02	0.98
After treatment	Accuracy-go trail (%)	96.32	4.12	95.06	3.56	0.96	0.33
	Accuracy-no go trail (%)	89.82	4.82	81.56	4.57	5.20	<0.001^***^
	RT-go trail (ms)	370.21	38.67	372.65	35.98	0.19	0.84
	RT-no go trail (ms)	432.12	43.98	435.45	43.65	0.22	0.82

RT, reaction time. ^***^ *p* < 0.001.

### 3.3 fNIRS results: resting-state functional connectivity

Prior to treatment, no significant differences in resting-state functional connectivity (RSFC) were observed between the two groups (*p* > 0.05). Following treatment, however, both groups exhibited a significant increase in RSFC compared to pre-treatment levels (*p* < 0.05). Specifically, the treatment group demonstrated a markedly elevated functional connectivity strength in the left and right dorsolateral prefrontal cortices, as well as in the left and right medial prefrontal cortices, when compared to the control group (*p* < 0.05). Conversely, no statistically significant differences were noted in the functional connectivity strength of the left and right temporal lobes between the two groups, as detailed in Table 4.

**Table 4:** Resting-state functional connectivity.

		Treatment group		Control group		*t*	*p*
		Mean	SD	Mean	SD		
Before treatment	LDLPFC	0.16	0.03	0.15	0.02	1.15	0.25
	RDLPFC	−0.04	0.01	−0.03	0.02	1.85	0.07
	LmPFC	−0.17	0.06	−0.16	0.05	0.53	0.59
	RmPFC	0.17	0.03	0.18	0.04	0.83	0.41
	LTL	0.17	0.05	0.18	0.06	0.53	0.59
	RTL	0.21	0.07	0.20	0.08	0.39	0.69
	All channels	0.09	0.03	0.08	0.02	1.16	0.25
After treatment	LDLPFC	0.32	0.08	0.25	0.03	3.46	<0.001^***^
	RDLPFC	0.26	0.05	0.19	0.03	5.05	<0.001^***^
	LmPFC	0.31	0.07	0.23	0.03	4.43	<0.001^***^
	RmPFC	0.42	0.12	0.29	0.09	3.63	<0.001^***^
	LTL	0.24	0.15	0.23	0.09	0.24	0.81
	RTL	0.29	0.12	0.28	0.03	0.34	0.73
	All channels	0.36	0.15	0.27	0.09	2.16	0.03^*^

LDLPFC, left dorsolateral prefrontal cortex; RDLPFC, right dorsolateral prefrontal cortex; LmPFC, left medial prefrontal cortex; RmPFC, right medial prefrontal cortex; LTL, left temporal lobe; RTL, right temporal Lobe. ^*^ *p* < 0.05, ^***^ *p* < 0.001.

### 3.4 fNIRS results: average HbO2 changes during the Go/no-go task

**Figure 3:**
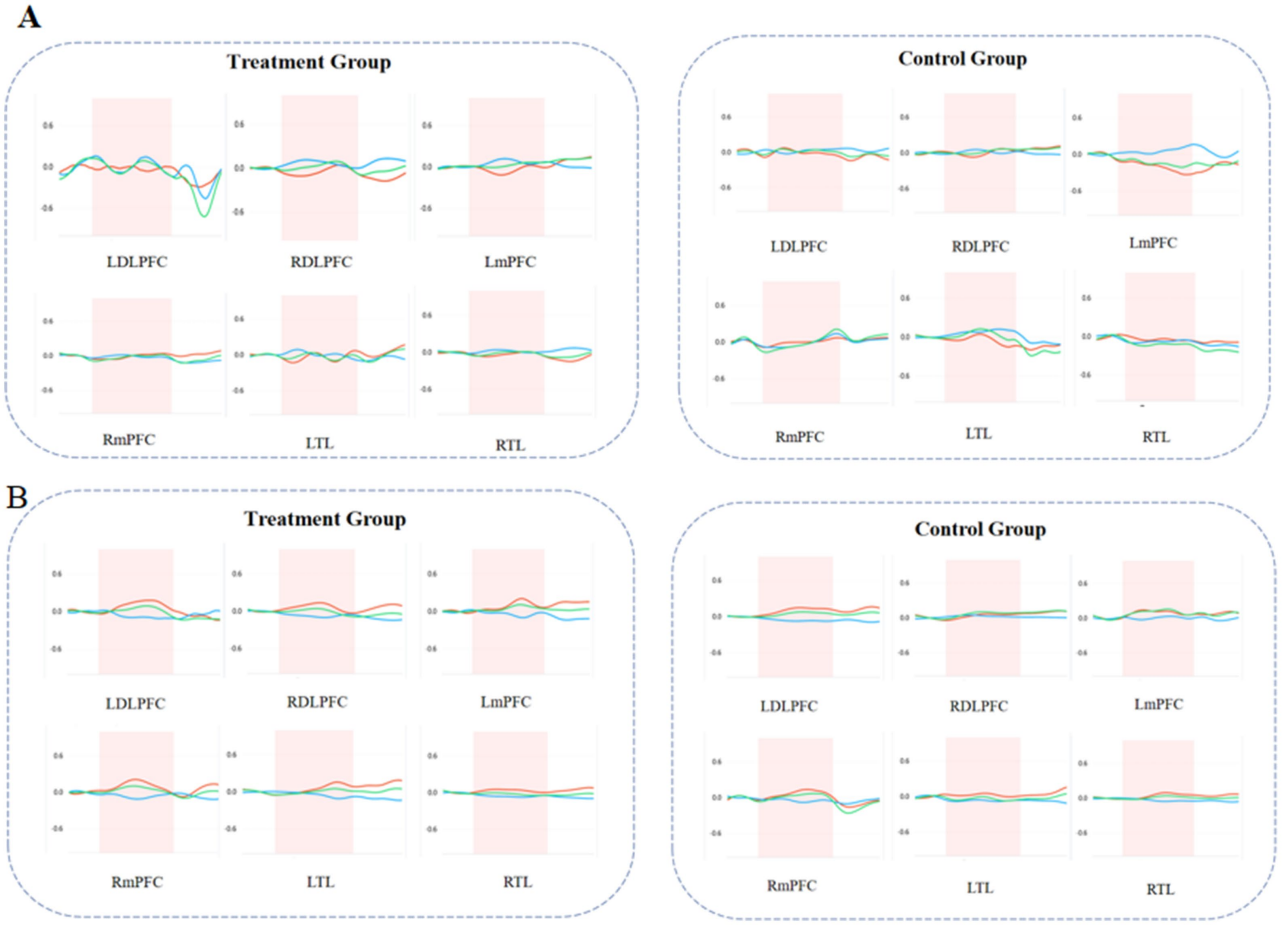
The HbO_2_ change curves of each brain region of subjects in the two groups during the Go/no-go task. **(A)** Before treatment. **(B)** After treatment. The red curve is the HbO_2_ change curves. LDLPFC, left dorsolateral prefrontal cortex; RDLPFC, right dorsolateral prefrontal cortex; LmPFC, left medial prefrontal cortex; RmPFC, right medial prefrontal cortex; LTL, left temporal lobe; RTL, right temporal Lobe.

In the Go/no-go task, the average HbO_2_ in the left dorsolateral prefrontal cortex, right dorsolateral prefrontal cortex, left medial prefrontal cortex, and right medial prefrontal cortex of the treatment group was significantly higher than that of the control group (*p* < 0.05). There was no statistically significant difference in the average HbO_2_ between the two groups in the left temporal lobe and right temporal lobe, as shown in Table 5 and Figure 3.

**Table 5:** Average HbO_2_ changes during the Go/no-go task.

		Treatment group		Control group		*t*	*p*
		Mean	SD	Mean	SD		
Before treatment	LDLPFC	0.13	0.03	0.12	0.02	1.16	0.25
	RDLPFC	−0.15	0.13	−0.14	0.12	0.23	0.81
	LmPFC	−0.09	0.03	−0.08	0.04	0.83	0.41
	RmPFC	0.14	0.05	0.16	0.07	0.96	0.34
	LTL	0.13	0.06	0.14	0.07	0.45	0.65
	RTL	0.17	0.08	0.18	0.11	0.30	0.76
After treatment	LDLPFC	0.37	0.12	0.29	0.08	2.33	0.02^*^
	RDLPFC	0.16	0.12	0.09	0.05	2.27	0.02^*^
	LmPFC	0.28	0.08	0.17	0.07	4.33	<0.001^***^
	RmPFC	0.39	0.12	0.24	0.09	4.20	<0.001^***^
	LTL	0.24	0.11	0.22	0.09	0.59	0.55
	RTL	0.36	0.13	0.34	0.09	0.53	0.59

LDLPFC, left dorsolateral prefrontal cortex; RDLPFC, right dorsolateral prefrontal cortex; LmPFC, left medial prefrontal cortex; RmPFC, right medial prefrontal cortex; LTL, left temporal lobe; RTL, right temporal Lobe. ^*^ *p* < 0.05, ^***^ *p* < 0.001.

### 3.5 Safety

No participants reported severe adverse events such as epileptic seizures or behavioral problems during the study session.

## 4 Discussion

This study applies fNIRS to investigate the impact of rTMS on the core symptoms and brain functional connectivity and activation in children with ADHD. Our findings provide valuable insights into the potential therapeutic benefits of rTMS in modulating the brain activity and alleviating ADHD symptoms. Our study results indicate that after treatment, the SNAP-IV scale scores of children in both groups decreased compared to before, with the treatment group scoring lower than the control group. This suggests that both rTMS in conjunction with non-pharmacological interventions and non-pharmacological interventions alone can improve the clinical symptoms of ADHD, with the combined treatment showing superior effects. This is essentially similar to the conclusion shown in related research (21) that the combination of non-pharmacological interventions with rTMS can enhance the therapeutic efficacy in children with ADHD. ADHD is a neurodevelopmental disorder characterized by core symptoms related to executive function deficits, including response inhibition and working memory. The incidence of ADHD is increasing year by year, but the causes and mechanisms of the disease are not yet clear, and there is a lack of targeted treatment plans. Currently, the diagnosis of ADHD mainly relies on the diagnostic criteria in DSM-V, which is completed based on the clinical manifestations and behavioral assessment scales of children, and there is a certain degree of subjectivity, lacking effective objective indicators. Early and accurate diagnosis is of great significance for children with ADHD to receive timely and effective treatment. At present, the clinical treatment for ADHD is mainly focused on non-pharmacological interventions, as a non-invasive, safe, and painless physical therapy method, sends pulsed magnetic fields to the head, where an electric field is generated in the cerebral cortex by the magnetic field, causing neurons to depolarize, generate action potentials, and improve motor, sensory, or cognitive functions (22). It plays an important role in regulating neural activity excitability, promoting neural synapse development, and neural transmission of neurotransmitters, and has been applied in the treatment of neurological diseases. Studies have found that rTMS can effectively improve the clinical symptoms of ADHD, which is consistent with our research findings (23).

fNIRS is an emerging optical neuroimaging technology used for non-invasive measurement of changes in blood oxygen concentration related to brain functional activity. According to the neurovascular coupling mechanism, when neurons are active, HbO_2_ increases and HbR decreases. fNIRS is simple to operate, has strong resistance to interference, and is highly compatible (24). Compared to fMRI, fNIRS also has high temporal resolution. With these advantages, there is growing interest in using fNIRS to study neuropsychiatric disorders. Resting-state is a natural imaging paradigm of fNIRS, which is convenient to operate and suitable for pediatric patients. Studies have shown that fNIRS resting-state detection can reveal changes in brain network during normal development and under psychopathological conditions, and can be used to identify brain network abnormalities in children with ADHD. In our study, we used fNIRS to collect the differences in blood oxygen concentration in the resting state of two groups of ADHD children and calculated the RSFC between the two groups. Post-intervention, both groups showed an increase in RSFC within the prefrontal cortex, with the treatment group exhibiting superior connectivity compared to the control group. This suggests that rTMS may enhance the functional integration of the prefrontal cortex, a region critical for executive functions such as attention, impulse control, and cognitive flexibility, which is often impaired in ADHD. The significant improvements in RSFC observed in the treatment group are consistent with the hypothesis that rTMS can modulate the neural networks behind these functions. The treatment group showed particularly pronounced RSFC in the left and right DLPFC and medial prefrontal cortex (mPFC), areas known to be involved in cognitive control and behavioral inhibition. This finding is significant because it suggests that rTMS may have targeted effects on neural circuits in ADHD. The absence of significant changes in the temporal lobes may indicate that rTMS exerts its primary effects on the prefrontal regions, which are more directly implicated in the pathophysiology of ADHD.

One of the core deficits of ADHD is the impairment of executive function response inhibition, and the executive dysfunction in children with ADHD is considered to be closely related to the prefrontal cortex. Response inhibition (such as the Go/no-go task) is one of the many tasks that distinguish ADHD patients from typically developing (TD) patients (25, 26). Therefore, many researchers have studied the response inhibition function of ADHD using the Go/no-go task paradigm. In our study, we used the fNIRS method to explore the differences in cortical brain function connectivity and activation between the two groups of children during the Go/no-go task. There were no significant differences in reaction time and accuracy in the Go block, and reaction time in the No-go block between the two groups of ADHD children. The treatment group had significantly lower accuracy in the Go/no-go block compared to the control (*p* < 0.05). During the Go/no-go task, the treatment group had higher HbO_2_ concentration and greater brain activation than the control, with more pronounced activation in the dorsolateral prefrontal cortex and medial prefrontal cortex, which is similar to the results reported in previous studies (27). During the Go/no-go task, the treatment group showed higher mean HbO_2_ concentrations in the prefrontal cortical regions, indicating increased neural activity and potentially better task performance. This is consistent with the observed improvements in RSFC and supports the idea that rTMS can enhance cognitive control and inhibitory processes, which is crucial for successful task performance in the Go/no-go paradigm.

rTMS offers a potential alternative or adjunctive treatment option for ADHD, particularly for those who do not respond well to or cannot tolerate pharmacological interventions. The non-invasive nature of rTMS and its potential to target specific cognitive deficits makes it a valuable area of research in the field of ADHD treatment. rTMS shows promise as a treatment for ADHD by modulating prefrontal cortical activation. fNIRS provides a valuable method for assessing these effects, offering insights into the neurobiological mechanisms underlying rTMS therapy. Future research should focus on optimizing rTMS protocols and exploring the durability of treatment effects. While these findings are promising, it is important to note that the therapeutic effects of rTMS may depend on the brain region targeted. Furthermore, the overall evidence for rTMS in ADHD treatment is still emerging, and more extensive clinical trials with larger sample sizes are needed to confirm these preliminary results and to explore the long-term efficacy and optimal treatment protocols for ADHD patients.

In conclusion, our study provides evidence that rTMS can significantly improve ADHD symptoms and enhance prefrontal cortical activation and connectivity in children with ADHD. These findings underscore the potential of rTMS as a non-invasive treatment option for ADHD and warrant further investigation into its therapeutic mechanisms and optimal treatment protocols.

## 5 Limitations

While our findings are promising, several limitations should be acknowledged. The relatively small sample size may limit the generalizability of our results, and a larger, more diverse sample would be necessary to confirm these findings. Additionally, the cross-sectional nature of this study does not allow for conclusions about the long-term effects of rTMS. Future research should employ longitudinal designs to assess the durability of treatment effects.

Moreover, the specific mechanisms by which rTMS exerts its effects on prefrontal cortical activation remain to be fully elucidated. Future studies should incorporate advanced neuroimaging techniques and explore potential biomarker to better understand the neurobiological changes associated with rTMS treatment.

## Data Availability

The original contributions presented in the study are included in the article/supplementary material, further inquiries can be directed to the corresponding author.
